# Books and Magazines

**Published:** 1913-10

**Authors:** 


### Books and Magazines.

HOW TO COLLECT A DOCTOR BILL.—By Frank P. Davis, M. D., 98 pages. Cloth bound. Price $1.00. Physicians Drug News, publishers, Newark, N. J.

This book is scarcely worth while. If a man makes up his mind to "beat" you, he'll do it. The way to collect your bill is to present it in person and if your party is honest and able to pay, now or later, he will do it. If not, destroy the bill and say no more about it.

---

EPISCOPAL HOSPITAL REPORTS.—Hospital of the Protestant Episcopal Church. Philadelphia, Vol. I. Press of W. J. Dornan, Philadelphia, 1913.

We are indebted to Dr. Artley P. C. Ashhurst, for this volume. He requests us to review it. As a rule hospital reports are rather dry reading, being made up, as a general thing, of figures and statistics; but any doctor, young or old, who takes any interest in his practice, will find pleasure and profit in reading this book. Here is embodied the vast clinical experience of a staff of the most distinguished physicians and surgeons in Philadelphia, in one of the most noted hospitals in America, in almost every department of medicine and surgery, clearly given in the text and beautifully illustrated by a number of illustrations drawn from life. It is a splendid handbook of clinical teaching. No doubt Dr. Ashhurst will send you a copy if you ask for it, naming this notice.

---

SYPHILIS AND THE NERVOUS SYSTEM for practitioners, Neurologists and Syphilologists.—By Max Nonne, Chief of the Nervous Department in the General Hospital, Hamburg, Eppendorf. Authorized translation from the second revised and enlarged German edition, by Charles R. Ball, B. A., M. D., Chief of the Nervous and Mental Department, St. Paul Free Dispensary; neurologist, St. Joseph Hospital, Bethesda Hospital, Mounds Park Hospital, Minnesota Soldiers Home and State Home for Crippled and Deformed Children. 98 illustrations in text. J. B. Lippincott Company, Philadelphia and London, 1913. Price $4.

Every doctor realizes the necessity of a late work on this subject. Developments in the study of syphilis have been so numerous and come so fast, as to be confusing and one must read up to keep up with them. In this work he will find them all, up-to-date. It is a necessity to the doctor who would keep informed.

BLOOD PRESSURE IN GENERAL PRACTICE.—By Percy Nicholson, M. D. Seven illustrations. J. B. Lippincott Company, Philadelphia and London. Price $1.50.

The subject of blood pressure, its significance, and its clinical uses as a diagnostic measure, is of recent introduction into the literature of medicine. Many of my readers doubtless know little or nothing of it except perhaps what they have read in scattered articles in an occasional journal. Here is a manual that will tell you all about it. Dr. Nicholson assumes that he is dealing with a new subject, and in writing this little book, he begins at the beginning and having reviewed all the available literature on the subject, has utilized such material as he deemed of clinical value, "avoiding what are, as yet, purely theoretic findings."

---

MALARIA: ETIOLOGY, PATHOLOGY, DIAGNOSIS, PROPHYLAXIS AND TREATMENT.—By Graham E. Henson, M. D., with an introduction by Charles C. Bass, M. D., Professor of Experimental Medicine, Tulane. C. V. Mosby Company, St. Louis. Price $2.50. Twenty-seven illustrations.

Dr. Henson has tackled a big subject and has condensed it into an octavo volume of 175 pages. It needs no review or criticism at my hands; the title tells the tale. I will only say that the problem of the eradication of malaria is now up to the sanitarian, and now that we know about the anopheles, it looks as if we might do that; the British have exterminated him and all of his kin, in the Blue Nile in the Soudan, and Gorgas has about gotten rid of him on the Isthmus. By the bye, let's call the disease something else than "malaria." Bad air has nothing to do with it.

---

DISEASES OF CHILDREN.—Henry Enos Tuley, M. D., late Professor of Obstetrics, University of Louisville, etc. 106 engravings and three colored plates including one (plate I, frontispiece) showing the Koplik Spots in Buccal Exanthem in Measles. The publishers say: "This is the first illustration, to our knowledge, showing Koplik's Spots." C. V. Mosby Company publishers, St. Louis, 1913. Price $5.50.

This is the second edition of Tuley's popular Diseases of Children. Many chapters have been entirely rewritten, and many additions to the text have been made, because of the advancements and discoveries in pediatrics since the appearance of the first edition in 1909. It is a complete text book for the student as well as a reliable and down-to-date book of reference for the general practitioner and the specialist. Mechanically, it is a credit to the publishers.

